# Supplementary material for: Engineering Saccharomyces cerevisiae for growth on xylose using an oxidative pathway
Source: Appl Microbiol Biotechnol. 2025 Jan 28;109(1):30. doi: 10.1007/s00253-025-13417-1 (PMC11775059; doi:10.1007/s00253-025-13417-1)
Supplement: Supplementary file 2 — Supplementary file2 (PDF 410 KB) [file 253_2025_13417_MOESM2_ESM.pdf]

Supplementary information for

Engineering *Saccharomyces cerevisiae* for growth on xylose using an oxidative pathway

Authors:

Kenya Tanaka[1,2,3,] Takahiro Yukawa[2], Takahiro Bamba[1,2], Miho Wakiya[2], Ryota Kumokita[2], Yong-Su Jin[4], Akihiko Kondo[1,2,5], Tomohisa Hasunuma[1,2,5*]

1 Engineering Biology Research Center, Kobe University, 1-1 Rokkodai, Nada, Kobe 657-8501, Japan

2 Graduate School of Science, Innovation and Technology, Kobe University, 1-1 Rokkodai, Nada, Kobe 657-8501, Japan;

3 Research Center for Solar Energy Chemistry, Graduate School of Engineering Science, Osaka University, 1-3 Machikaneyama, Toyonaka, Osaka, 560-8531, Japan

4 Carl Woese Institute for Genomic Biology, University of Illinois at Urbana-Champaign, Urbana, IL, USA

5 Research Center for Sustainable Resource Science, RIKEN, Yokohama, Kanagawa 230-0045, Japan

*Corresponding author

Tomohisa Hasunuma -- Email: hasunuma@port.kobe-u.ac.jp; Phone: +81-78-803-6356; Fax: +81-78-803-6192

Supplemental Figure

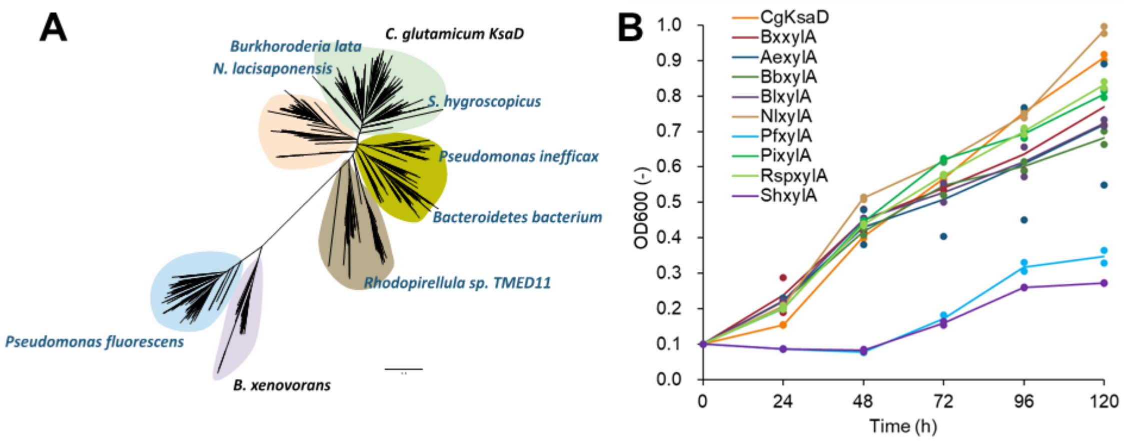

Supplemental Fig. 1 XylA screening based on phylogenetic tree analysis. (A) Phylogenetic tree analysis of XylA; (B) Yeast growth tests were performed on medium containing 20 g/L of xylose as the sole carbon source. The line graph shows the means of individual results (dots) obtained from two independent experiments.

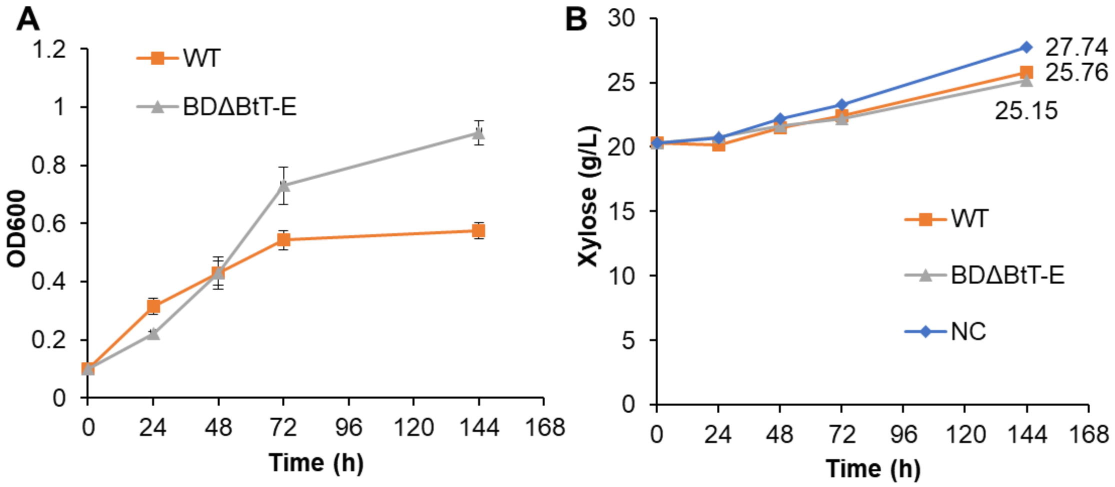

Supplemental Fig. 2 Additional data on growth on xylose as the sole carbon source via the Dahms pathway (A) and the time course of xylose concentration (B) are presented. The xylose concentration in the medium without cells is shown as the negative control (NC).

Supplemental Table 1. Plasmids used in this study

Plasmid name	Description
pTS-A-xylBD	pTDH3-xylB, pSED1-xylD, ADE2 marker
pIAur-tTYW1	PGK1p-tTYW1-PGK1t, AUR1-C marker
pIL-pTDH3 tADH1	pTDH3-tADH1, LEU2 marker
pIL-pTDH3-yagE	pTDH3-yagE-tADH1, LEU2 marker
pIU-pTDH3-CgksaD	pTDH3-CgksaD-tADH1, URA3 marker
pIL-pSED1-tSAG1	pSED1p-tSAG1, LEU2 marker
pIL-pSED1-HaxylX-tSAG1	pSED1p-HaxylX-tSAG1, LEU2 marker
pIL-pSED1-HpxylX-tSAG1	pSED1p-HpxylX-tSAG1, LEU2 marker
pIL-pSED1-CcxylX-tSAG1	pSED1p-CcxylX-tSAG1, LEU2 marker
pIL-pSED1-BxxylX-tSAG1	pSED1p-BxxylX-tSAG1, LEU2 marker
pIL-pSED1-NtxylX-tSAG1	pSED1p-NtxylX-tSAG1, LEU2 marker
pIL-pSED1-SaxylX-tSAG1	pSED1p-SaxylX-tSAG1, LEU2 marker
pIA-pTDH3-tADH1	pTDH3-tADH1, ADE2 marker
pIA-pTDH3-xylB	pTDH3-xylB-tADH1, ADE2 marker
pGK406	Empty vector, URA3 marker
pIU-pTDH3-tADH1	pTDH3-tADH1, URA3 marker
pIU-pTDH3-BxxylA	pTDH3-BxxylA-tADH1, URA3 marker
pIU-pTDH3-AexylA	pTDH3-AexylA-tADH1, URA3 marker
pIU-pTDH3-ShxylA	pTDH3-ShxylA-tADH1, URA3 marker
pIU-pTDH3-PixylA	pTDH3-PixylA-tADH1, URA3 marker
pIU-pTDH3-BbxylA	pTDH3-BbxylA-tADH1, URA3 marker
pIU-pTDH3-RspxylA	pTDH3-RspxylA-tADH1, URA3 marker
pIU-pTDH3-PfxylA	pTDH3-PfxylA-tADH1, URA3 marker
pIU-pTDH3-SaxylA	pTDH3-SaxylA-tADH1, URA3 marker
pIU-pTDH3-RbxylA	pTDH3-RbxylA-tADH1, URA3 marker
pIU-pTDH3-BlxylA	pTDH3-BlxylA-tADH1, URA3 marker
pIU-pSED1-tSAG1	pSED1p-tSAG1, URA3 marker
pIU-pSED1-MaxylD	pSED1p-MaxylD-tSAG1, URA3 marker
pIU-pSED1-CcxylD	pSED1p-CcxylD-tSAG1, URA3 marker
pIU-pSED1-BxxylD	pSED1p-BxxylD-tSAG1, URA3 marker
pIU-pSED1-HexylD	pSED1p-HexylD-tSAG1, URA3 marker
pIU-pSED1-SexylD	pSED1p-SexylD-tSAG1, URA3 marker

pIU-pSED1-AtxylD	pSED1p-AtxylD-tSAG1, URA3 marker
pIU-pSED1-RmxylD	pSED1p-RmxylD-tSAG1, URA3 marker
pIU-pSED1-PaxylD	pSED1p-PaxylD-tSAG1, URA3 marker

Supplemental Table 2. Primers used in this study

Primer	Sequence
xhoI-SED1p F	cgggcccccccctcgagattggatatagaaaattaacgtaagg
xhoI-SED1p R	aactgtacacccgggcttaatagagcgaacgtattttattttg
SAG1t F	cccgggtgtacagttagtacattgagtc
SAG1t R	accgcggtggcggccgcatccagtgagcgcgcgtaatacgac
dBOL2_ ADE2 F	ttcttgaataatacataacttttc
dBOL2_ ADE2 R	ataagtgatcttatgtatgaaattc
BOL2 up F	acgttctctccgttgttcaaacccc
BOL2 up R	agttatgtattattcaagaaatatatgtatatatataacaccg
BOL2 down F	tcatacataagatcacttataaaggatgatattgttctattattaag
BOL2 down R	acagcaacgacgacaatgccaaacc

Supplemental Table 4. Yeast strains constructed in this study

Strain name	Description
YPH499	MATa ura3-52 lys2-801_amber ade2-101_ochre trp1-Δ63 his3-Δ200 leu2-Δ1
YPH499ΔGRE3	YPH499, gre3Δ::kanMX4
BD	YPH499ΔGRE3, pTS-A-xylBD
BDE	BD, pIL-TDH3p-yagE
BDΔB-E	BD, bol2Δ::HIS3, pIL-TDH3p-yagE
BDΔBtT	BD, bol2Δ::HIS3, pIAur-tTYW1
BDΔBtT-E	BDΔBtT, pIL-TDH3p-yagE
BDΔBtT-H	BDΔBtT, pIL-TDH3p-yagE
BE	YPH499ΔGRE3, pIA-pTDH3-xylB, pIL-TDH3p-yagE
BEΔBtT	BE, bol2Δ::HIS3, pIAur-tTYW1
BEΔBtT-MaxylD	BEΔBtT, pIU-pSED1-MaxylD
BEΔBtT-CcxylD	BEΔBtT, pIU-pSED1-CcxylD
BEΔBtT-BxxylD	BEΔBtT, pIU-pSED1-BxxylD
BEΔBtT-HexylD	BEΔBtT, pIU-pSED1-HexylD
BEΔBtT-SexylD	BEΔBtT, pIU-pSED1-SexylD
BEΔBtT-AtxylD	BEΔBtT, pIU-pSED1-AtxylD
BEΔBtT-RmxylD	BEΔBtT, pIU-pSED1-RmxylD
BEΔBtT-PaxylD	BEΔBtT, pIU-pSED1-PaxylD
BDΔBtT-ksaD	BDΔBtT, pIU-pTDH3-CgksaD
BDΔBtT-ksaD-CcxylX	BDΔBtT-ksaD, pIL-pSED1-CcxylX-tSAG1
BDΔBtT-ksaD-HaxylX	BDΔBtT-ksaD, pIL-pSED1-HaxylX-tSAG1
BDΔBtT-ksaD-HpxylX	BDΔBtT-ksaD, pIL-pSED1-HpxylX-tSAG1
BDΔBtT-ksaD-BxxylX	BDΔBtT-ksaD, pIL-pSED1-BxxylX-tSAG1
BDΔBtT-ksaD-SaxylX	BDΔBtT-ksaD, pIL-pSED1-SaxylX-tSAG1
BDΔBtT-ksaD-NtxylX	BDΔBtT-ksaD, pIL-pSED1-NtxylX-tSAG1
BDΔBtT-BxxylX	BDΔBtT, pIL-pSED1-BxxylX-tSAG1
BDΔBtT-BxxylX-BxxylA	BDΔBtT-BxxylX, pIU-pTDH3-BxxylA
BDΔBtT-BxxylX-AexylA	BDΔBtT-BxxylX, pIU-pTDH3-AexylA
BDΔBtT-BxxylX-ShxylA	BDΔBtT-BxxylX, pIU-pTDH3-ShxylA
BDΔBtT-BxxylX-PixylA	BDΔBtT-BxxylX, pIU-pTDH3-PixylA
BDΔBtT-BxxylX-BbxylA	BDΔBtT-BxxylX, pIU-pTDH3-BbxylA

BDΔBtT-BxxylX-RspxylA	BDΔBtT-BxxylX, pIU-pTDH3-RspxylA
BDΔBtT-BxxylX-PfxylA	BDΔBtT-BxxylX, pIU-pTDH3-PfxylA
BDΔBtT-BxxylX-SaxylA	BDΔBtT-BxxylX, pIU-pTDH3-SaxylA
BDΔBtT-BxxylX-RbxylA	BDΔBtT-BxxylX, pIU-pTDH3-RbxylA
BDΔBtT-BxxylX-BlxylA	BDΔBtT-BxxylX, pIU-pTDH3-BlxylA
